# The MAGIC trial: a pragmatic, multicentre, parallel, noninferiority, randomised trial of melatonin *versus* midazolam in the premedication of anxious children attending for elective surgery under general anaesthesia^☆^

**DOI:** 10.1016/j.bja.2023.10.011

**Published:** 2023-11-10

**Authors:** Robert Bolt, Marie C. Hyslop, Esther Herbert, Diana E. Papaioannou, Nikki Totton, Matthew J. Wilson, Janet Clarkson, Christopher Evans, Nicholas Ireland, Jennifer Kettle, Zoe Marshman, Amy C. Norrington, Robert H. Paton, Christopher Vernazza, Christopher Deery, the MAGIC collaborative, Sondos Albadri, Laura Armstrong, Simon Atkins, Margaret Babb, Claire Biercamp, Katie Biggs, Mike Bradburn, Jaimie Buckley, Julie Child-Cavill, Sean Cope, Simon Crawley, Munya Dimairo, Enass Duro, Ayman Eissa, Laura Flight, Jacqui Gath, Gil Gavel, Tim Geary, Fiona Gilchrist, Padma Gopal, Jamie Hall, Kate Hutchence, Puran Khandelwal, Pranav Kukreja, Ian Leeuwenberg, James Limb, Amanda Loban, Katie Mellor, Nuria Masip, Anthony Moores, Vimmi Oshan, Edward Pickles, Jaydip Ray, Helen Rodd, Sian Rolfe, Elena Sheldon, Richard Simmonds, Rachel Smith, Ashok Sundar, Anna Thomason, Simon Waterhouse, Graham Wilson, Julian Yates, Tracey Young

**Affiliations:** 1 School of Clinical Dentistry, University of Sheffield, Sheffield, UK; 2 Sheffield Clinical Trials Research Unit, ScHARR, University of Sheffield, Sheffield, UK; 3 Sheffield School of Health & Related Research, University of Sheffield, Sheffield, UK; 4 Dundee Dental Hospital and School, University of Dundee, Dundee, UK; 5 University College London Hospitals NHS Foundation Trust, London, UK; 6 Newcastle upon Tyne Hospitals NHS Foundation Trust, Newcastle upon Tyne, UK; 7 South Tees Hospitals NHS Foundation Trust, Middlesbrough, UK; 8 Barnsley Hospital NHS Foundation Trust, Barnsley, UK; 9 School of Dental Sciences, Newcastle University, Newcastle upon Tyne, UK

**Keywords:** general anaesthesia, melatonin, midazolam, paediatric anxiety, perioperative care, premedication

## Abstract

**Background:**

Child anxiety before general anaesthesia and surgery is common. Midazolam is a commonly used premedication to address this. Melatonin is an alternative anxiolytic, however trials evaluating its efficacy in children have delivered conflicting results.

**Methods:**

This multicentre, double-blind randomised trial was performed in 20 UK NHS Trusts. A sample size of 624 was required to declare noninferiority of melatonin. Anxious children, awaiting day case elective surgery under general anaesthesia, were randomly assigned 1:1 to midazolam or melatonin premedication (0.5 mg kg^−1^, maximum 20 mg) 30 min before transfer to the operating room. The primary outcome was the modified Yale Preoperative Anxiety Scale-Short Form (mYPAS-SF). Secondary outcomes included safety. Results are presented as *n* (%) and adjusted mean differences with 95% confidence intervals.

**Results:**

The trial was stopped prematurely (*n*=110; 55 per group) because of recruitment futility. Participants had a median age of 7 (6–10) yr, and 57 (52%) were female. Intention-to-treat and per-protocol modified Yale Preoperative Anxiety Scale-Short Form analyses showed adjusted mean differences of 13.1 (3.7–22.4) and 12.9 (3.1–22.6), respectively, in favour of midazolam. The upper 95% confidence interval limits exceeded the predefined margin of 4.3 in both cases, whereas the lower 95% confidence interval excluded zero, indicating that melatonin was inferior to midazolam, with a difference considered to be clinically relevant. No serious adverse events were seen in either arm.

**Conclusion:**

Melatonin was less effective than midazolam at reducing preoperative anxiety in children, although the early termination of the trial increases the likelihood of bias.

**Clinical trial registration:**

ISRCTN registry: ISRCTN18296119.

**Editor’s key points**

• Anxiolysis for children undergoing short day-case surgical procedures is an important intervention.
• The findings of this randomised trial suggest melatonin is less effective than midazolam for preoperative anxiolysis in children.
• However, the trial was terminated early because of recruitment fatigue, which makes the findings more susceptible to bias.

Each year, ∼487,000 children undergo general anaesthesia in the UK.^1^ Perioperative anxiety in children and their accompanying caregivers ahead of anaesthesia and surgery is common, with up to 50% of children exhibiting distress behaviour at the point of anaesthetic induction.^2^^,^^3^ High levels of preoperative anxiety have been linked to non-compliance in the anaesthetic room, abandonment, and subsequent rescheduling of surgery, and increased risk of adverse postoperative outcomes such as pain, emergence delirium, and delayed behavioural changes.^2^^,^^4^, ^5^, ^6^, ^7^ Current methods to alleviate anxiety in children include non-pharmacological interventions such as play therapy, distraction techniques, and interactive games, alongside a heterogeneous range of anxiolytic (and often sedative) premedications. In the UK, those children with high levels of anxiety are recommended a premedication to reduce distress ahead of surgery.^2^ Common premedication regimes include single dose or combination dosing of midazolam, clonidine, ketamine or dexmedetomidine.^8^

Midazolam is a benzodiazepine and remains the most commonly prescribed oral premedication in the UK for anxious children before general anaesthetic.^9^ Although effective at reducing anxiety, it has undesirable side-effects including respiratory depression, oversedation/delayed postoperative recovery, disorientation, agitation, and delirium.^2^^,^^10^, ^11^, ^12^ Concerns have been raised with respect to the long-term impact of sedative medications in young children, as their repeated use has sometimes been correlated with the subsequent manifestation of learning disabilities.^13^ Melatonin is a native hormone involved in the regulation of the human circadian rhythm. It acts through multiple pathways to regulate the sleep–wake cycle and enhances somnolence.^14^ The drug is licensed in the paediatric setting for management of sleep onset insomnia and delayed sleep phase syndrome, although it is often used off licence in the management of sleep disturbances relating to learning disabilities and behavioural challenges.^15^ Melatonin has been shown to be an effective premedication before surgery in adults, with a Cochrane systematic review concluding that the drug may be as effective as midazolam at reducing preoperative anxiety.^14^ Despite evidence of melatonin’s effectiveness in the adult population, studies in children have delivered conflicting results.^2^ A systematic review of preoperative melatonin use in children was unable to confirm whether the drug was comparable to standard premedications, including midazolam.^16^ Few adverse events (AE) were found to be attributable to melatonin, confirming the drug’s excellent safety profile in the paediatric setting. Several limitations of the included studies were reported, notably that trials took place in a general paediatric preoperative population rather than selecting for specifically anxious children.

The aim of the MAGIC trial was to evaluate the effectiveness of melatonin compared with midazolam in the premedication of anxious children before general anaesthesia with non-inferiority methodology. The rationale for completing this comparison on a non-inferiority basis is represented by the improved AE profile of melatonin. Secondary trial objectives include the comparison of side-effects observed with each drug and the time to discharge.

## Methods

### Study design and participants

This multicentre, parallel-group, double blind, individual participant-randomised trial took place across 20 UK NHS Hospital Trusts. Delegated research staff identified, assented, and randomised paediatric participants identified as anxious on the day of surgery. Trial information was given via age-specific patient information sheets and short video animation. Eligible participants were anxious children aged 3–14 yr, scheduled for elective dental, ear, nose, and throat (ENT), ophthalmology, gastroenterology, radiology, plastic, orthopaedic, urology, or general surgery under general anaesthetic. As a pragmatic trial, preoperative anxiety as eligibility for inclusion was left to clinical judgement of the responsible anaesthetist, as per local standard of care. This reflects normal practice in the UK. There is no clinically validated objective measure or universal guidance on the selection of children for premedication in the UK, where the decision is based on anaesthetists' judgement. Therefore, prescribing practice can vary from clinician to clinician. Decision-making for premedication is undertaken at preassessment clinics, on the morning of surgery when the anaesthetist assesses the child, or both. Participants were American Society of Anesthesiologists (ASA) physical status 1 or 2 and required written caregiver’s consent for entry into the trial.

Exclusion criteria were: children deemed non-anxious by the responsible anaesthetist; surgery requiring inpatient admission (non-day-case); premedication required for reasons other than anxiety; current prescription of melatonin, midazolam, or drug that contraindicated the co-prescription of either trial medication; presence of severe learning disabilities rendering verbal and written communication difficult; ASA physical status 3, 4, or 5; and caregiver not consenting to participation in the trial. This population was similar to that of trials which assessed the efficacy of midazolam.^10^

### Randomisation and blinding

Participants were randomly assigned in a 1:1 ratio to either control (midazolam) or treatment (melatonin) arms. Randomisation was computer-generated and completed using minimisation based on centre, surgical speciality (head and neck, gastroenterology and MRI, and other) and sex (male/female). All hospitals involved in the trial were assessed before inclusion by a pre-trial audit and feasibility assessment to ensure comparability, including case lists, anaesthetic teams, use of paediatric wards/recovery facilities, and midazolam prescribing practices. Hospitals were similar in that they were either teaching hospitals or large district general hospitals serving comparable general populations, with appropriate dedicated paediatric lists from which patients were further carefully specified by means of the trial’s inclusion/exclusion criteria.

The trial was double-blind, with the participant, clinical care team, and assessor (research nurse or trained member of clinical staff) all blinded to treatment allocation. Allocation concealment was achieved using a centralised web-based randomisation system, with treatment allocation revealed solely to an unblinded trial pharmacist for drug dispensation. Trial medication for both arms was supplied in matching single-use plastic bottles of identical dosage strengths, raspberry flavourant, and volume. There was a risk that the staff member administering the Investigational Medicinal Product (IMP) may become unblinded; either through observing the child’s taste reaction as a result of midazolam being associated with unfavourable taste/rejection^17^; or as a result of the visible signs linked to midazolam’s sedative effect.^2^ The IMP was administered by a separate nurse who was not involved in outcome assessments.^2^ All participants were transferred to theatre via trolley with the benefit of concealing the sedative effects of midazolam.

### Data collection

A series of anxiety and recovery measures, for both child participants and their caregivers, were administered on the day of surgery (pre- and post-surgery) and 14 days post-discharge. Figure S1 in Supplementary material (File 2) outlines the participant journey, stages of assessment, and time points at which data were gathered throughout the trial (including State Trait Anxiety Inventory [STAI] questionnaire^18^; Cooperation Score^19^; Quality of Life Child Health Utility 9D [QoL CHU9D] questionnaire^20^; Revised Faces Pain Scale [FPS-R observer and participant reported]^21^; Paediatric Anaesthesia Emergence Delirium scale [PAED] index^22^; Vancouver Sedation Recovery Scale [VSRS]^23^; and Post Hospitalization Behaviour Questionnaire for Ambulatory Surgery [PHBQ-AS]^24^). Participants were cared for on a dedicated paediatric ward by dedicated paediatric staff. Modified Yale Preoperative Anxiety Scale (mYPAS) assessors were paediatric nursing staff, paediatric research nurses, or anaesthetists, all of whom had received appropriate training and had relevant expertise and experience.

### Interventions

Participants were allocated to receive either melatonin or midazolam 0.5 mg kg^−1^, with a maximum allowed dose of 20 mg, similar to other trials which assessed the efficacy of midazolam.^2^ The blinded IMP was administered orally as a single measured dose via syringe, 30 min before transfer to theatre by a trained healthcare professional. If the participant rejected the premedication because of taste, a suspected unblinding was recorded. In cases of rejection, the responsible anaesthetist determined whether re-dosing was required. Participants who required re-dosing were withdrawn from the trial. All formal and suspected cases of staff unblinding were recorded (See Supplementary material (File 3)).

### Primary outcome measure—modified Yale Preoperative Anxiety Scale-Short Form

The primary endpoint was modified Yale Preoperative Anxiety Scale-Short Form (mYPAS-SF) score (adjusted for baseline) measured over the three consecutive, standard preoperative time points recommended for the scale: start of transfer to theatre, entry into anaesthetic room, and administration of anaesthesia. The mYPAS-SF is the current gold standard for assessing child anxiety during induction of anaesthesia.^25^^,^^26^ Assessors received training to minimise inter-examiner variability via an e-learning package developed by University College London (UCL, Little Journey trial).^27^ Further details on the training are provided in Supplementary material (File 4).

### Secondary outcome measures

A selection of secondary outcome measures were recorded to further assess participant and caregiver preoperative anxiety and postoperative child recovery, including; anaesthetic failure, Cooperation Score, FPS-R observer and participant reported, caregiver STAI questionnaire, VSRS, and PAED index. At 2 weeks post-surgery, longer-term measures of anaesthetic impact were assessed using the PHBQ-AS and QoL CHU9D.

### Sample size

The sample size, based on mYPAS-SF over all three time points whilst adjusting for baseline (assumed correlation 0.5),^28^ was 624 participants. This assumed 90% power, 2.5% one-sided alpha, standard deviation of 25,^29^ 5% attrition and a non-inferiority margin of 4.3. A noninferiority margin of 4.3 was established using preceding work by Jenkins and colleagues,^26^ who found a clinically meaningful difference to be 12.9 on the mYPAS scale. One-third of the score was agreed a suitable non-inferiority margin.^30^

### Statistical analysis

All of the statistical analysis was performed according to the MAGIC Statistical Analysis Plan^31^ which was written before the decision to close the study early because of recruitment futility. Participants were included in intention-to-treat (ITT) analyses if they had completed baseline mYPAS-SF and at least one follow-up mYPAS-SF measure. The per-protocol (PP) analysis population comprised all randomised participants who took a complete dose of study drug within the allowed time window (20–75 min pre-transfer), with no major protocol deviations. Participants were analysed based on the treatment they were randomised to in both analysis populations. The primary outcome was analysed using a linear mixed effects model with treatment, follow-up time point, baseline score, sex, surgical speciality group and centre as fixed effects, and participant as a random intercept. Non-inferiority would be declared if the upper limit of the 95% confidence interval (CI) of the adjusted mean difference (melatonin *vs* midazolam) did not exceed the predefined non-inferiority margin of 4.3.^2^ Other continuous, longitudinal secondary outcomes were analysed in the same way. The number and proportion of participants experiencing at least one AE were summarised for each treatment group. AEs were recorded for all participants who had received at least some of the study drug and were allocated on the basis of treatment received. The statistical analysis is reported according to CONSORT guidelines extension for pragmatic and noninferiority trials.^2^^,^^32^

### Approvals

The full MAGIC trial protocol is available via the University of Sheffield data repository, ORDA.^2^ Patient and public involvement (PPI) was incorporated throughout the trial, in both the design phase and study implementation. Protocol amendments during the trial are provided in Supplementary Table S1 in Supplementary material (File 5). The trial was approved by Liverpool Central NRES Committee (REC reference 18/NW/0758) and received Medicines and Healthcare products Regulatory Agency (MHRA) approval (21304/0267/001-0001). Sheffield Clinical Trials Research Unit (CTRU) coordinated follow-up and data collection in collaboration with the trial centres.

## Results

### Early closure

The MAGIC trial opened to recruitment in July 2019. In early 2020, the COVID-19 pandemic severely disrupted the trial, which was halted and then reopened to recruitment in October 2020, closed again in February 2021, and re-opened again in June 2022. However, the ongoing pandemic led to challenges in drug supply, paediatric elective surgery list recovery (post-COVID), and therefore trial recruitment. With agreement from the trial steering committee and the trial funders, the trial was terminated early in November 2022, on the grounds of recruitment futility. A total of 110 participants were recruited to the trial; the original sample size was 624. Barriers to the recruitment of anxious children undergoing day-case surgery are discussed elsewhere.^33^

### Recruitment, randomisation, and withdrawal

**Fig 1:**
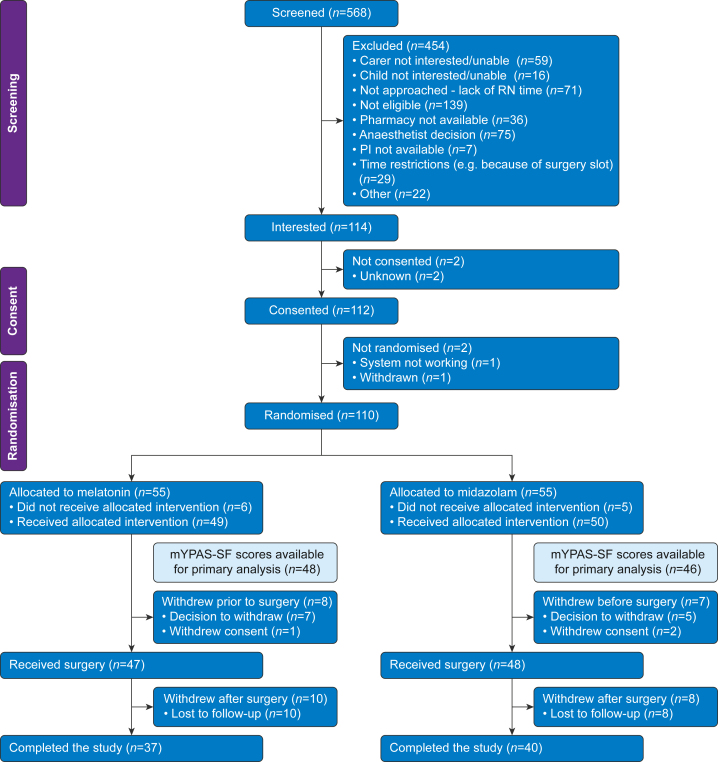
CONSORT flow diagram. mYPAS-SF, modified Yale Preoperative Anxiety Scale-Short Form; PI, principal investigator; RN, research nurse.

**Fig 2:**
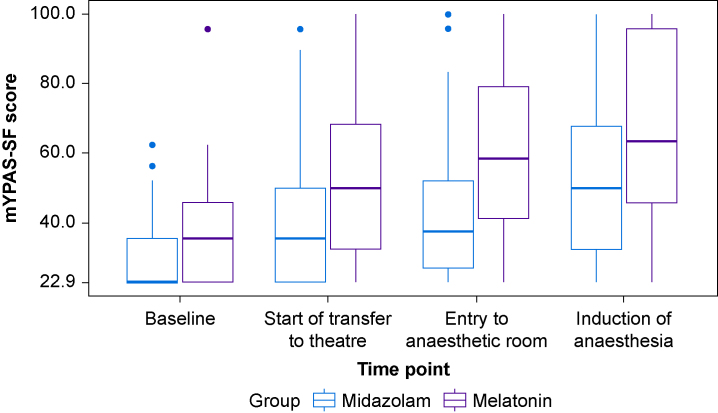
Boxplots of mYPAS-SF scores at each time point by treatment group. mYPAS-SF, modified Yale Preoperative Anxiety Scale-Short Form.

Between July 30, 2019 and November 9, 2022, 110 participants were randomised across 17 centres; 55 to receive melatonin and 55 to receive midazolam (Fig. 1). Baseline characteristics for these participants are summarised in Table 1. The mean age of participants was 7.9 yr (standard deviation [sd] 2.6). Median baseline mYPAS-SF was 29.2 (inter-quartile range [IQR] 22.9–45.8) with a chance imbalance between the two arms. Figure 1 illustrates a CONSORT flow diagram of recruitment into the trial. Fifteen participants withdrew post-randomisation; reasons are summarised in Supplementary Table S2 provided in Supplementary material (File 6). A further 18 participants were lost to follow-up at 14 days. For the latter group all preoperative and immediate postoperative measures, including mYPAS-SF scores, were collected with high fidelity. Preoperative reasons for withdrawal classed as ‘other’ included unacceptable pharmacy delays in IMP dispensation and participant refusal to take the IMP. For withdrawn participants, where available, data were included in the analyses. The IMP was administered to 90% of participants (99/110). Taste rejection of IMP occurred in two (4%) cases in the midazolam arm and four (7%) cases in the melatonin arm. The IMP was administered outside of the recommended window (20–75 min) in two participants, both in the melatonin arm (Fig. 2).

**Table 1:** Baseline characteristics of randomised participants. Select percentages may not sum to 100 because of rounding. Assessed numbers are given where data were not available for the whole population. ENT, ear, nose, and throat; IQR, inter-quartile range; mYPAS-SF, modified Yale Preoperative Anxiety Scale-Short Form; STAI, State Trait Anxiety Inventory.

		Midazolam	Melatonin
		*N*=55	*N*=55
Age, yr	Median (IQR)	8.0 (6.0–10.0)	7.0 (6.0–9.0)
Sex, *n* (%)	Male	26 (47)	27 (49)
	Female	29 (53)	28 (51)
Height, cm	*n*	33	41
	Median (IQR)	130.0 (119.0–146.0)	132.0 (118.0–144.0)
Weight, kg	*n*	54	55
	Median (IQR)	29.5 (21.6–42.2)	27.7 (22.1–40.8)
Baseline mYPAS-SF scor	*n*	53	55
	Mean (sd)	30.54 (11.01)	37.80 (14.68)
Ethnicity, *n* (%)	English/Welsh/Scottish/Northern Irish/British	52 (95)	50 (91)
	Indian	0 (0)	1 (2)
	Pakistani	0 (0)	1 (2)
	White and black Caribbean	0 (0)	1 (2)
	White and Asian	0 (0)	1 (2)
	Any other white background	1 (2)	0 (0)
	African	0 (0)	1 (2)
	Missing	2 (4)	0 (0)
Surgical speciality, *n* (%)	Dental	39 (71)	33 (60)
	ENT	8 (15)	8 (15)
	Ophthalmology	7 (13)	11 (20)
	Gastroenterology	0 (0)	1 (2)
	Urology	0 (0)	1 (2)
	Other general surgery, specify	1 (2)	1 (2)
ASA physical status, *n* (%)	ASA 1	44 (80)	45 (82)
	ASA 2	10 (18)	10 (18)
	Missing	1 (2)	0 (0)
Caregiver STAI S-anxiety	n	54	53
	Median (IQR)	39.0 (30.2–47.0)	42 (29.0–48.0)
Caregiver STAI T-anxiety	*n*	55	56
	Median (IQR)	38 (27.5–45.0)	34.0 (27.8–45.2)

### Primary outcome measure (mYPAS-SF)

**Fig 3:**
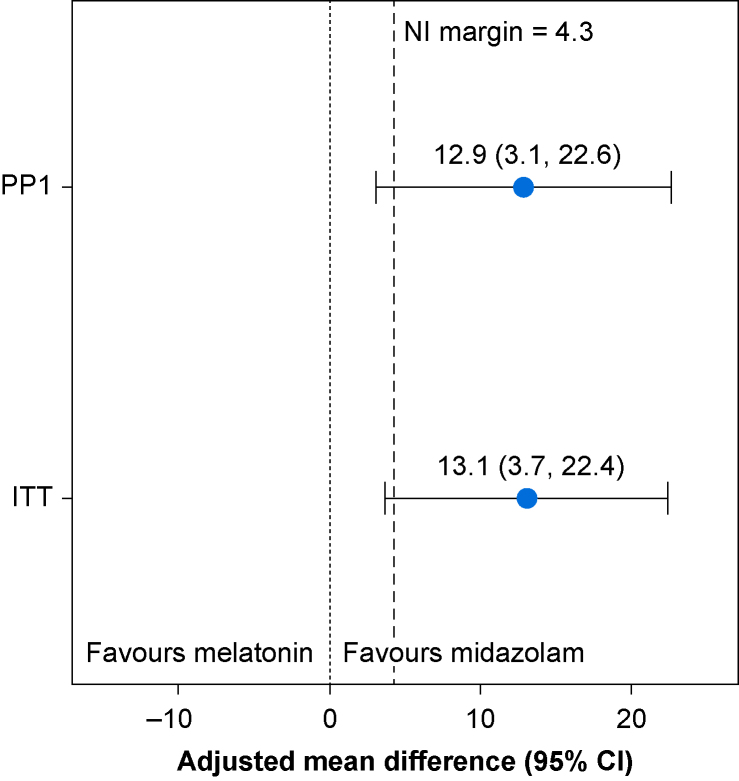
Adjusted mean difference in mYPAS-SF of melatonin compared with midazolam from the primary analysis models. (Mean [standard deviation]) mYPAS-SF scores and adjusted mean differences from the primary analysis model for each analysis population located in Supplementary Table S5 provided in Supplementary material (File 6). ITT, intention-to-treat; mYPAS-SF, modified Yale Preoperative Anxiety Scale-Short Form; NI, noninferiority; PP, per-protocol.

The primary outcome measure, mYPAS-SF, was completed at baseline and at least one follow-up time point for 94 participants (86%), with all data included in the ITT analysis. Six participants (6%) were excluded from the PP analysis. The adjusted mean difference in mYPAS-SF scores for melatonin *vs* midazolam was 13.1 (95% CI 3.7–22.4) in favour of midazolam for the ITT population and 12.9 (95% CI 3.1–22.6) for the PP population (Fig. 3). In both analyses, the upper and lower limits of the 95% CI exceeded the prespecified non-inferiority margin (4.3), providing evidence that melatonin was inferior to midazolam in reducing preoperative anxiety in the trial population (Fig. 3).

### Secondary outcome measures

Full results from all of the secondary outcomes can be found in Supplementary Table S3 and S4 provided in Supplementary material (File 6). Three anaesthetic failures occurred (two melatonin arm, one midazolam arm). Median anaesthetic turnaround time was 43 min (IQR: 27–65 min). A log transformation was used when fitting the analysis model, because of the skewness of the data. The adjusted mean ratio was 1.01 min (95% CI 0.8–1.3 min) for melatonin compared with midazolam for the ITT analysis population.

Post-surgery, PAED index decreased over time from a mean score of 11.6 (sd: 3.1) at 15 min to 2.2 (sd: 3.3) at 2 h across all participants. The number of participants with available scores also decreased over time (from 79 to 32) because of hospital discharge readiness. Joint modelling of PAED index with time to discharge readiness shows an adjusted mean difference over time of −0.70 (95% CI −2.04 to 0.64) in favour of midazolam compared with melatonin.

VSRS scores increased over time from a mean score of 13.7 (sd: 5.0) at 15 min to 20.4 (sd: 3.4) at 2 h. However, as with PAED index, the number of participants with available scores also decreased (from 35 to 27). Joint modelling of VSRS score with time to discharge readiness shows an adjusted mean difference of 0.12 (95% CI −1.57 to 1.81) in favour of melatonin compared with midazolam (summarised in Supplementary Table S3 provided in Supplementary material (File 6)).

### Adverse events

Of the participants receiving the IMP (99/110, 90%), 22 (22%) had at least one AE (Table 2). More AEs were seen in the midazolam arm compared with the melatonin arm (23 *vs* 11). Most AEs were mild (grade 1) (30/34; 88%) and required no action (21/34; 62%). There were no serious AE (SAEs) reported for the trial.

**Table 2:** Number of adverse events and proportion of participants with at least one adverse event.

		Midazolam		Melatonin	
		Events	Individuals	Events	Individuals
			*N*=50		*N*=49
All adverse events, *n* (%)		23	13 (26)	11	9 (18)
Intensity, *n* (%)	Mild	22	13 (26)	8	7 (14)
	Moderate	1	1 (2)	3	3 (6)
Relationship to study drug, *n* (%)	Reasonable possibility of being related	1	1 (2)	0	0 (0)
	No reasonable possibility of being related	17	11 (22)	8	6 (12)
	Not assessable	5	4 (8)	3	3 (6)
Action taken, *n* (%)	None	14	10 (20)	7	6 (12)
	Other	6	5 (10)	1	1 (2)
	Specific treatment	3	3 (6)	3	3 (6)
Outcome, *n* (%)	Recovered	20	12 (24)	11	9 (18)
	Not recovered	2	2 (4)	0	0 (0)
	Recovered with sequelae	1	1 (2)	0	0 (0)
Preferred term, *n* (%)	Agitation	0	0 (0)	1	1 (2)
	Anxiety	2	2 (4)	0	0 (0)
	Bradypnoea	1	1 (2)	0	0 (0)
	Emotional distress	1	1 (2)	2	2 (4)
	Epistaxis	1	1 (2)	1	1 (2)
	Flushing	1	1 (2)	0	0 (0)
	Haemorrhage	2	2 (4)	1	1 (2)
	Hypotension	2	2 (4)	0	0 (0)
	Infection	0	0 (0)	1	1 (2)
	Nausea	1	1 (2)	0	0 (0)
	Nightmare	1	1 (2)	0	0 (0)
	Pain	5	5 (10)	3	3 (6)
	Syncope	1	1 (2)	0	0 (0)
	Tachypnoea	1	1 (2)	0	0 (0)
	Vomiting	3	3 (6)	2	2 (4)
	Wound dehiscence	1	1 (2)	0	0 (0)
System organ class, *n* (%)	Gastrointestinal disorders	4	4 (8)	2	2 (4)
	General disorders and administration site conditions	4	4 (8)	3	3 (6)
	Infections and infestations	0	0 (0)	1	1 (2)
	Injury, poisoning, and procedural complication	1	1 (2)	0	0 (0)
	Investigations	1	1 (2)	0	0 (0)
	Nervous system disorders	1	1 (2)	0	0 (0)
	Psychiatric disorders	4	4 (8)	3	3 (6)
	Respiratory, thoracic, and mediastinal disorders	3	3 (6)	1	1 (2)
	Vascular disorders	5	5 (10)	1	1 (2)

## Discussion

Here we report the first multicentre, randomised trial of melatonin *vs* midazolam to be undertaken in a specifically anxious child population before general anaesthesia. A marked decrease in the primary outcome measure, adjusted mYPAS-SF, was observed in the midazolam group, refuting non-inferiority between groups and showing inferiority of melatonin compared with midazolam as an anxiolytic premedication in children. There was a chance imbalance in baseline mYPAS-SF scores in favour of midazolam; the analysis accounted for any bias this may have introduced by looking at adjusted scores rather than raw score, ensuring that the observed inferiority of melatonin relative to midazolam is a reliable finding. Moreover, the trial protocol pre-specifying the use of adjusted mYPAS-SF as a primary outcome measure rather than absolute mYPAS-SF value, is less susceptible to the baseline fluctuation in mYPAS-SF encountered between groups. Reassuringly, despite the chance variation in baseline mYPAS-SF scores, all other baseline characteristics of trial participants, along with potential confounders such as post-randomisation trial withdrawals, were comparable in both melatonin and midazolam arms (Table 1 and provided in Supplementary material (File 6)).

The magnitude of difference in adjusted mYPAS-SF observed in the midazolam group compared with melatonin was a consistent finding throughout all three time points (transfer, entry into anaesthetic room, and anaesthetic induction). Consequently, both ITT and PP analyses delivered 95% CI heavily skewed in favour of midazolam (Fig. 3), with both adjusted mean difference and upper CI limits well exceeding the prespecified non-inferiority margin of 4.3, and lower CI fully excluding zero. Therefore, despite limitations in recruitment, we conclude a clinically meaningful^26^ difference in effectiveness of midazolam compared with melatonin. This finding conflicts with a number of previous, smaller trials,^34^, ^35^, ^36^, ^37^, ^38^ and is likely accounted for by the fact that only specifically anxious children were recruited to this trial. Conversely, previous trials that included non-anxious child participants are likely to have introduced a significant source of dilution of true effect size in both midazolam and melatonin groups, accounting for the observation of drug comparability.

**Fig 4:**
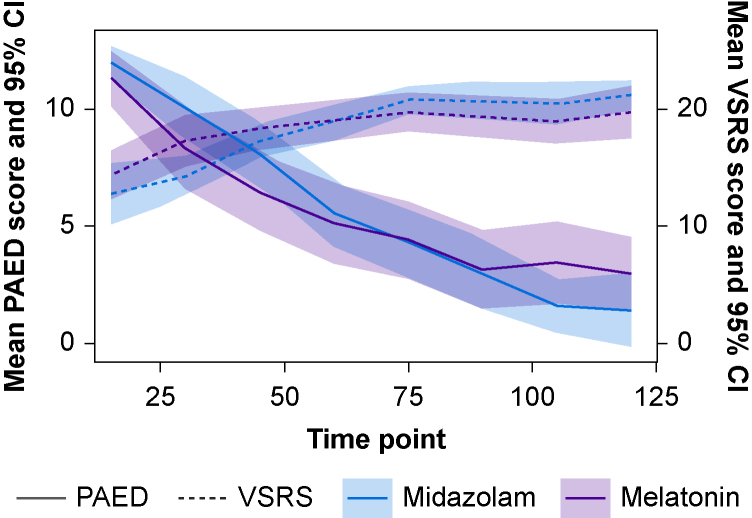
Mean post-surgery PAED and VSRS scores (with 95% CI) by treatment group. CI, confidence interval; PAED, Paediatric Anaesthesia Emergence Delirium scale; VSRS, Vancouver Sedation Recovery Scale.

The MAGIC trial gathered a number of secondary outcomes to assist with triangulation of those properties of a favourable premedication that may not be directly linked to anxiolytic efficacy. Regarding all secondary outcome measures, our findings are underpowered and so care should be taken with their interpretation. Midazolam’s clinical profile led to a pre-trial assumption that postoperative recovery would be more impaired in comparison to the melatonin group, although trial measures of sedation recovery (VSRS) and emergence delirium (PAED index) demonstrated no such difference between groups (Fig. 4). No increase in anaesthetic recovery time was observed in midazolam compared with melatonin, challenging a common-held assumption that premedicating with midazolam leads to delayed recovery following-general anaesthesia. Indeed, the limited available literature suggests only minor delays in the order of 4–10 min.^11^ There were marginally more participants with at least one AE in the midazolam arm (*n*=13) compared with melatonin (*n*=9), with only one AE (nightmares) potentially related to the IMP (midazolam). There were no SAEs in either arm. These secondary outcome measures, in combination with AE and SAE data, suggest that potential disadvantages of midazolam occur infrequently. Although midazolam was more effective at reducing anxiety than melatonin, it should be noted that anaesthetic failure rates were low in both groups (midazolam *n*=1: melatonin *n*=2). There were also comparable incidences of anaesthetist decision to re-dose because of inadequate anxiolysis before transfer, suggesting that both drugs had beneficial effect. As discussed, although the trends in these secondary measures are of interest, the CI were wide, which limits their interpretation. The trial was also limited by exclusively using the MYPAS-SF measure solely as the primary outcome, as opposed to other common measures of sedation (e.g. mask acceptance or level of restraint).

A number of barriers to recruitment were noted during a formal qualitative analysis, undertaken as part of the internal pilot of the trial^33^ 2021. Many of these barriers are unlikely to have influenced the generalisability of the trial results, as they related to resource pressures such as lack of research nurse availability. However, certain resource pressures, such as research pharmacies not being open at the commencement of operating lists, could have impacted more significantly on the first patient of the respective theatre list. A common anaesthetic practice is to list more challenging patients at the start of a given theatre list and therefore these patients may not have been recruited. Trial randomisation acts to account for such confounders by distributing the confounders equally between trial arms.

### Future studies

The trial showed that using melatonin as a premedication was less effective at reducing preoperative anxiety than midazolam, further adding to the knowledge base within the perioperative anaesthetic field of effective anxiolytic paediatric premedications. However, given the findings of the MAGIC trial, there still remains a clinical need to develop or repurpose another premedication with a more favourable side-effects profile. A recent 2022 systematic review evaluated the sedative effects of various commonly used premedications, including: dexmedetomidine, midazolam, clonidine, ketamine, and melatonin, in managing preoperative anxiety in children (aged <7 yr).^39^ They reported no significant difference in sedative effect or mask acceptance between melatonin and placebo, with superiority seen in the other four premedications. Furthermore, they found midazolam did not reduce emergence delirium and prolonged length of PACU stay compared with placebo; and use of midazolam, ketamine, and clonidine lead to several side-effects. Based on side-effect profiles, this systematic review concluded that dexmedetomidine may be a more optimal sedative for premedication in children. An increasing use of dexmedetomidine amongst UK paediatric anaesthetists was frequently noted during the MAGIC trial, and further research into this drug as a more suitable alternative premedication could be beneficial.

### Conclusions

The trial did not reach the required sample size and therefore is prone to bias. Within the population studied, melatonin is less effective than midazolam at reducing preoperative anxiety in children and young people before general anaesthesia. The current standard of care, midazolam, was better by a clinically meaningful margin.

## Authors' contributions

CD (chief investigator), RB (co-investigator), MCH (trial manager), EH (statistician), DP (CTRU lead), NT (statistician) and MW (co-investigator) together produced the first draft of the manuscript. All members of the authorship group, provided in Supplementary material (File 1), read and approved the final manuscript, offering critical feedback.

Conceived of or designed the work: RB, DP, CD, NT, MW, ZM, JC, SoA, SA, MB, JB, JCC, AE, FG, JR, HR, GW, JY, TY on behalf of the MAGIC collaborative.

Involved in the acquisition of data for the work: MCH, ES, AL, RS, JH, KH, AT, KM, SW and CD on behalf of the MAGIC collaborative.

Involved in the analysis of data: EH, NT, MB, AL, RS, RB, DP, CD, MW, MCH on behalf of the MAGIC collaborative.

Responsible for statistical analysis and modelling: EH, NT, MB on behalf of the MAGIC collaborative (Supplementary material (File 1)).

Responsible for project level steering, national coordination, and data collection: the trial steering committee and data monitoring and ethics committee.

Responsible for ensuring adherence to hospital-level governance protocols and regional data collection: local leads.

Revised the work critically for important intellectual content: all authors.

Involved in the final approval of the version to be published: all authors.

## Declaration of interest

The authors declare that they have no conflicts of interest.

## Funding

The MAGIC trial was funded by the NIHR Health Technology Assessment programme (NIHR HTA 16/80/08). Further information available at: https://fundingawards.nihr.ac.uk/award/16/80/08.
