# Supplementary material for: The MAGIC trial: a pragmatic, multicentre, parallel, noninferiority, randomised trial of melatonin versus midazolam in the premedication of anxious children attending for elective surgery under general anaesthesia
Source: Br J Anaesth. 2023 Nov 10;132(1):76–85. doi: 10.1016/j.bja.2023.10.011 (PMC10797512; doi:10.1016/j.bja.2023.10.011)
Supplement: Multimedia component 1 [file mmc1.pdf]

Appendix A - Supplementary Data File 1

Authors & Collaborators

MAGIC Authors Writing Group: Robert Bolt, Marie C Hyslop, Esther Herbert, Diana E Papaioannou, Nikki Totton, Matthew J Wilson, Janet Clarkson, Christopher Evans, Nicholas Ireland, Jennifer Kettle, Zoe Marshman, Amy C Norrington, Robert H Paton, Christopher Vernazza and Christopher Deery

MAGIC Collaborator Group (excluding authors): Sondos, Albadri, Laura Armstrong, Simon Atkins, Margaret Babb, Claire Biercamp, Katie Biggs, Mike Bradburn, Jaimie Buckley, Julie Child-Cavill, Sean Cope, Simon Crawley, Munya Dimairo, Enass Duro, Ayman Eissa, Laura Flight, Jacqui Gath, Gil Gavel, Tim Geary, Fiona Gilchrist, Padma Gopal, Jamie Hall, Kate Hutchence, Puran Khandelwal, Pranav Kukreja, Ian Leeuwenberg, James Limb, Amanda Loban, Katie Mellor, Nuria Masip, Anthony Moores, Vimmi Oshan, Edward Pickles, Jaydip Ray, Helen Rodd, Sian Rolfe, Elena Sheldon, Richard Simmonds, Rachel Smith, Ashok Sundar, Anna Thomason, Simon Waterhouse, Graham Wilson, Julian Yates and Tracey Young.
