# Supplementary material for: The MAGIC trial: a pragmatic, multicentre, parallel, noninferiority, randomised trial of melatonin versus midazolam in the premedication of anxious children attending for elective surgery under general anaesthesia
Source: Br J Anaesth. 2023 Nov 10;132(1):76–85. doi: 10.1016/j.bja.2023.10.011 (PMC10797512; doi:10.1016/j.bja.2023.10.011)
Supplement: Multimedia component 2 [file mmc2.pdf]

Appendix A - Supplementary Data File 2

MAGIC Participant Pathway

Figure S1. MAGIC participant pathway

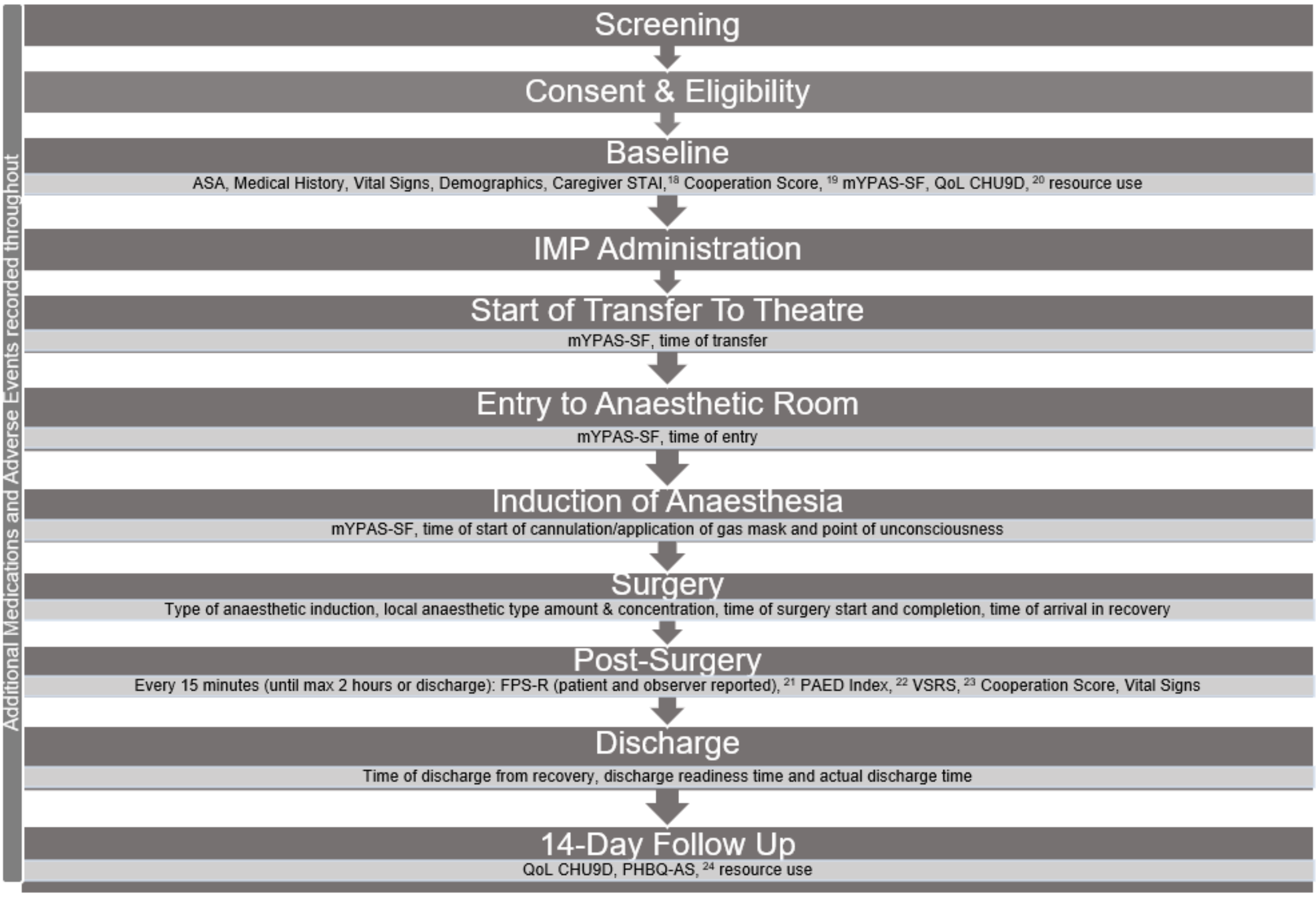

State Trait Anxiety Inventory (STAI) questionnaire;[18] *Cooperation Score;* [19] *Quality of Life Child Health Utility 9D (QoL CHU9D) questionnaire;*[20] *Revised Faces Pain Scale (FPS-R observer and participant reported);*[21] *Paediatric Anaesthesia Emergence Delirium scale (PAED) index;*[22] *Vancouver Sedation Recovery Scale (VSRS;)* [23] *Post Hospitalization Behaviour Questionnaire for Ambulatory Surgery (PHBQ-AS).* [24]
