# Supplementary material for: The MAGIC trial: a pragmatic, multicentre, parallel, noninferiority, randomised trial of melatonin versus midazolam in the premedication of anxious children attending for elective surgery under general anaesthesia
Source: Br J Anaesth. 2023 Nov 10;132(1):76–85. doi: 10.1016/j.bja.2023.10.011 (PMC10797512; doi:10.1016/j.bja.2023.10.011)
Supplement: Multimedia component 3 [file mmc3.pdf]

Appendix A - Supplementary Data File 3

Unblinding

Any instances of suspected unblinding were recorded, including the reasons for and time point of unblinding. The observer research nurse and anaesthetist were asked to complete a short data collection form which recorded the following:

a) either personnel believe they have been unblinded
b) the reason for unblinding, for example, how the participant behaved
c) at what stage in the process of data collection unblinding occurred
d) the perceived group which the apparently unblinded child was allocated

This was monitored throughout the trial to inform trial feasibility. There were 9 cases of suspected unblinding reported, and 1 formal case (within 9 participants). In the 9 participants, 6 spat out IMP, 1 was formal unblinding and 3 were other.
