# Supplementary material for: The MAGIC trial: a pragmatic, multicentre, parallel, noninferiority, randomised trial of melatonin versus midazolam in the premedication of anxious children attending for elective surgery under general anaesthesia
Source: Br J Anaesth. 2023 Nov 10;132(1):76–85. doi: 10.1016/j.bja.2023.10.011 (PMC10797512; doi:10.1016/j.bja.2023.10.011)
Supplement: Multimedia component 4 [file mmc4.pdf]

# Appendix A - Supplementary Data File 4

## MYPAS-SF training

The mYPAS-SF assessment was the primary outcome for the trial. The measure has been used in a wide range of studies and is considered the current gold-standard for assessing a child's anxiety during induction of anaesthesia.[1,2] The tool is observational and divided into four items: activity, vocalisations, emotional expressivity and state of apparent arousal; with each item having a Likert-type scale of options reflecting a child's behaviour, with higher scores indicating the highest severity within that item.[3]

As this was a judgement-based outcome, e-learning training was required for all assessors to minimise variation between individuals' assessments. There were no standardised methods to train independent observers to use the mYPAS-SF tool or defined required intra- or inter-rater reliability. The founder of the tool, Dr Zeev Kain, had used the tool in multiple single-centre studies since its conception quoting a variety of different methods of training and required inter-rater reliabilities of observers, with many requiring observers to achieve a set inter-rater reliability prior to being allowed to perform mYPAS-SF scoring.[1,2]

The mYPAS-SF requires a combination of both clinical and non-clinical training. The creation of an e-learning package by University College London (UCL - Little Journey trial[3]) enabled observers-in-training to complete the non-clinical, clinical and reliability testing phases through distance learning creating a consistent measurement approach. MAGIC assessors were required to achieve reliability levels of at least 0.70 ((kappa statistic) and were mandated to retrain every 12 months.

References (also in main text)


1. Kain ZN, Mayes LC, Cicchetti DV, Caramico LA, Spieker M, Nygren MM, Rimar S. Measurement tool for preoperative anxiety in young children: The yale preoperative anxiety scale. Child Neuropsychology. 1995; 1:3, 203-210, DOI: 10.1080/09297049508400225

2. Jenkins BN, Fortier MA, Kaplan SH, Mayes LC, Kain ZN. Development of a short version of the modified Yale Preoperative Anxiety Scale. Anesth Analg. 2014 Sep;119(3):643-650. doi: 10.1213/ANE.0000000000000350. PMID: 25010821.
3. Little Journey Trial: https://www.littlejourney.health/
