# Supplementary material for: The MAGIC trial: a pragmatic, multicentre, parallel, noninferiority, randomised trial of melatonin versus midazolam in the premedication of anxious children attending for elective surgery under general anaesthesia
Source: Br J Anaesth. 2023 Nov 10;132(1):76–85. doi: 10.1016/j.bja.2023.10.011 (PMC10797512; doi:10.1016/j.bja.2023.10.011)
Supplement: Multimedia component 5 [file mmc5.pdf]

Appendix A - Supplementary Data File 5

MAGIC Protocol Changes

Table S1. Summary of changes to the MAGIC trial protocol

**Changes to Protocol**	**Date**	**Approved by**
**Protocol version 2.0 (not implemented)**:<br>Updated in response to REC request to remove the £10 vouchers for qualitative study interviewees	19th December 2018	N/A
**Protocol version 3.0 (approved version on trial opening):**<br>Updates included change from PHBQ to PHBQ-AS; removal of post box test; update to non-permitted medication; change to allow verbal assent for all children; change to allow for nurse prescribers; change to allow for postal return CHU9D questionnaires at follow up; change of timing for post-operative assessments to every 15 mins from every 10 mins; removal of out of hours unblinding system; replacement of 'until stage 2 recovery completion'	27th March 2019	North West - Liverpool Central Research Ethics Committee<br><br>Health Research Authority<br><br>Medicines & Healthcare products Regulatory Agency
**Protocol version 4.0**:<br>Updates included change to expand inclusion criteria to allow 3 and 4 year old children to be recruited; change to expand inclusion criteria to include more surgical specialities: gastroenterology, radiology, plastic, orthopaedic, urology or other general surgery; change to clarify assent requirement from children. Children who neither provide assent nor decline the trial (due to high anxiety) can be enrolled based on caregiver consent and PI decision. Children who verbally decline to participate must not be included; change for those sites who do not have dedicated pre-operative clinics, to allow the team to send study information prior to the day of surgery. This will be based on PI decision of the suitability of the participant to receive this information; change to randomisation system from stratification to minimisation; change to allow children the option of reviewing the information sheet or the video and not a requirement to undertake both; clarification of secondary safety and efficacy objectives and outcomes and addition of CHU-9D proxy questionnaire for children aged 3-4.	7th May 2020	North West - Liverpool Central Research Ethics Committee<br><br>Health Research Authority<br><br>Medicines & Healthcare products Regulatory Agency
**Protocol version 4.1:**<br>Updates included change to clarify assent from highly anxious children not mandatory and can be based on caregiver and PI (or delegated individual) decision alone; change to clarify baseline assessments can be undertaken after randomisation; change to allow remote consent and interviews for the qualitative sub-study in light of the 2020 COVID-19 pandemic.	28th September 2020	Non substantial – no approvals required.
