# Supplementary material for: The MAGIC trial: a pragmatic, multicentre, parallel, noninferiority, randomised trial of melatonin versus midazolam in the premedication of anxious children attending for elective surgery under general anaesthesia
Source: Br J Anaesth. 2023 Nov 10;132(1):76–85. doi: 10.1016/j.bja.2023.10.011 (PMC10797512; doi:10.1016/j.bja.2023.10.011)
Supplement: Multimedia component 6 [file mmc6.pdf]

Appendix A - Supplementary Data File 6

Additional Results

Table S2. Trial withdrawals (post-randomisation) by time point.

		Midazolam N = 55	Melatonin N = 55
Between randomisation and surgery	Total	7 (13%)	8 (15%)
	Anaesthetist decision to re-dose	2/7 (29%)	2/8 (25%)
	Other	5/7 (71%)	6/8 (75%)
Between surgery and follow up	Total	8 (15%)	10 (18%)
	Lost to follow up	8/8 (100%)	10/10 (100%)

Table S3. Secondary outcomes and adjusted effect sizes with 95% CIs.

Outcome	Population	Time point	Midazolam n	Midazolam Mean (SD)	Melatonin n	Melatonin Mean (SD)	Adjusted effect sizes (95% CI)
Anaesthetic turnaround time (minutes)	ITT	-	46	46.2 (29.9)	41	51.5 (34.1)	1.01 (0.78, 1.3)[a]
	PP	-	45	46.8 (30.0)	35	53.4 (36.3)	1.02 (0.77, 1.36)[a]
VSRS	ITT	15	15	12.73 (5.19)	20	14.35 (4.79)	0.122 (-1.567, 1.811)[b]
		30	32	14.28 (4.89)	26	17.27 (5.77)	
		45	32	17.28 (4.10)	31	18.35 (5.08)	
		60	29	18.90 (4.39)	28	19.04 (4.86)	
		75	25	20.80 (2.87)	25	19.72 (4.27)	
		90	22	20.68 (3.86)	21	19.33 (3.97)	
		105	19	20.42 (3.99)	18	18.94 (4.12)	
		120	14	21.14 (2.41)	13	19.69 (4.15)	
	PP	15	15	12.73 (5.19)	18	14.50 (5.03)	0.413 (-1.331, 2.156)[b]
		30	31	14.29 (4.97)	22	18.09 (5.58)	
		45	31	17.13 (4.07)	27	18.89 (4.70)	
		60	28	18.79 (4.43)	23	19.91 (4.18)	
		75	24	20.75 (2.92)	21	19.95 (4.52)	
		90	21	20.62 (3.94)	18	19.67 (4.03)	
		105	19	20.42 (3.99)	15	18.93 (4.43)	
		120	14	21.14 (2.41)	10	19.00 (4.55)	
PAED	ITT	15	40	11.97 (2.36)	39	11.31 (3.74)	-0.699 (-2.039, 0.641)[b]
		30	43	10.05 (4.38)	38	8.32 (5.39)	
		45	38	8.08 (4.24)	38	6.45 (5.05)	
		60	30	5.53 (3.95)	34	5.12 (5.08)	
		75	31	4.29 (4.04)	32	4.41 (4.74)	
		90	25	2.96 (3.86)	25	3.16 (4.22)	
		105	20	1.60 (2.52)	20	3.45 (3.94)	
		120	15	1.40 (3.09)	17	2.94 (3.38)	
	PP	15	39	11.97 (2.39)	33	11.39 (3.77)	-0.856 (-2.167, 0.456)[b]
		30	42	10.05 (4.43)	33	7.97 (5.46)	

Outcome	Population	Time point	Midazolam n	Midazolam Mean (SD)	Melatonin n	Melatonin Mean (SD)	Adjusted effect sizes (95% CI)
		45	37	8.03 (4.28)	33	6.18 (5.20)	
		60	29	5.59 (4.01)	29	4.83 (5.20)	
		75	30	4.30 (4.11)	27	3.93 (4.71)	
		90	24	3.00 (3.93)	21	2.57 (3.78)	
		105	20	1.60 (2.52)	17	3.41 (4.21)	
		120	15	1.40 (3.09)	14	3.00 (3.55)	

[a] Adjusted mean ratio; [b] Adjusted mean difference

Table S4. Additional Secondary Outcomes not included in the main text

	Population & Time point		Midazolam n	Midazolam Mean (SD)	Melatonin n	Melatonin Mean (SD)	Adjusted Effect Size (95% CI)
Recovery time (minutes)	ITT		44	145.3 (74.2)	41	142.7 (70.2)	0.88 (0.74, 1.04)[a]
	PP		43	146.5 (74.6)	35	145.3 (67.6)	0.95 (0.8, 1.13)[a]
PHBQ	ITT		39	3.0 (0.4)	35	3.0 (0.3)	-0.03 (-0.21, 0.14)[b]
	PP		38	3.0 (0.4)	30	3.0 (0.3)	-0.08 (-0.27, 0.11)[b]
Analgesics required	ITT			45/55 (81.82%)		47/55 (85.45%)	1.22 (0.37, 4.25)[c]
	PP			44/47 (93.62%)		41/43 (95.35%)	0.75 (0.05, 19.31)[c]
Co-operation score	ITT	15	38	0.92 (2.02)	38	1.39 (2.57)	-0.064 (-0.843, 0.716)[b]
		30	43	3.02 (2.81)	35	3.26 (3.25)	
		45	37	4.16 (2.69)	36	4.64 (2.95)	
		60	30	5.37 (2.40)	34	5.06 (2.80)	
		75	31	5.90 (1.99)	31	5.32 (2.76)	
		90	25	6.16 (1.60)	24	6.00 (2.13)	
		105	20	6.40 (1.39)	21	6.24 (2.12)	
		120	15	6.27 (1.44)	17	6.53 (1.70)	
	PP	15	37	0.95 (2.04)	32	1.25 (2.44)	0.047 (-0.764, 0.858)[b]
		30	42	2.98 (2.82)	32	3.44 (3.29)	
		45	36	4.19 (2.72)	32	4.69 (3.05)	
		60	29	5.31 (2.42)	29	5.24 (2.82)	
		75	30	5.87 (2.01)	26	5.88 (2.41)	
		90	24	6.12 (1.62)	20	6.50 (1.61)	
		105	20	6.40 (1.39)	18	6.22 (2.26)	
		120	15	6.27 (1.44)	14	6.50 (1.87)	
FPS-R (child reported)	ITT	15	4	5.00 (4.76)	5	3.40 (3.97)	0.170 (-0.388, 0.729)[b]
		30	16	2.31 (3.46)	14	2.43 (3.25)	
		45	21	2.86 (3.44)	22	1.64 (2.11)	
		60	22	3.23 (3.54)	24	2.54 (3.53)	
		75	23	2.22 (2.59)	22	2.23 (3.12)	
		90	19	1.53 (2.32)	20	2.30 (3.13)	
		105	18	2.44 (3.11)	17	2.00 (2.45)	
		120	12	0.67 (1.30)	14	1.71 (2.20)	
	PP	15	4	5.00 (4.76)	4	1.75 (1.71)	0.126 (-0.419, 0.671)[b]
		30	15	2.47 (3.52)	14	2.43 (3.25)	

	Population & Time point		Midazolam		Melatonin		Adjusted Effect Size (95% CI)
			n	Mean (SD)	n	Mean (SD)	
		45	20	3.00 (3.46)	21	1.71 (2.12)	
		60	21	3.29 (3.62)	22	2.77 (3.60)	
		75	22	2.23 (2.65)	21	2.33 (3.15)	
		90	18	1.50 (2.38)	17	2.71 (3.24)	
		105	18	2.44 (3.11)	15	2.27 (2.49)	
		120	12	0.67 (1.30)	11	2.00 (2.37)	
FPS-R (observer reported)	ITT	15	27	2.44 (3.20)	29	1.14 (1.73)	-0.046 (-0.585, 0.493)[b]
		30	37	2.38 (2.75)	30	1.83 (2.49)	
		45	34	2.38 (2.93)	36	2.28 (2.87)	
		60	31	2.32 (2.37)	32	1.91 (3.03)	
		75	31	1.61 (2.09)	31	1.65 (2.80)	
		90	26	1.73 (2.25)	25	1.00 (1.91)	
		105	20	0.85 (1.35)	21	1.33 (2.24)	
		120	15	1.13 (1.81)	16	0.94 (1.77)	
	PP	15	27	2.44 (3.20)	24	1.12 (1.75)	0.013 (-0.610, 0.636)[b]
		30	36	2.44 (2.76)	26	2.04 (2.60)	
		45	33	2.45 (2.95)	31	2.58 (2.98)	
		60	30	2.33 (2.41)	27	2.26 (3.18)	
		75	30	1.60 (2.13)	26	1.96 (2.96)	
		90	25	1.72 (2.30)	21	1.19 (2.04)	
		105	20	0.85 (1.35)	18	1.56 (2.36)	
		120	15	1.13 (1.81)	13	1.15 (1.91)	

Table S5. Mean (SD) mYPAS-SF scores and adjusted mean differences from the primary analysis model for each analysis population.

Population	Treatment	Start of transfer to theatre		Entry to anaesthetic room		Induction of anaesthesia		Adjusted MD (95% CI)
		n	Mean (SD)	n	Mean (SD)	n	Mean (SD)	
ITT	Midazolam	44	40.5 (20.1)	44	43.1 (20.8)	46	51.7 (24.1)	13.1 (3.7, 22.4)
	Melatonin	48	51.2 (23.7)	48	60.3 (22.7)	48	66.1 (26.5)	
PP	Midazolam	44	41.4 (20.7)	44	43.2 (20.8)	46	51.0 (23.9)	12.9 (3.1, 22.6)
	Melatonin	43	52.3 (24.2)	43	61.0 (23.7)	43	66.8 (27.3)	
